# Supplementary material for: Long-term effects of early postnatal stress on Sertoli cells
Source: Front Genet. 2022 Oct 24;13:1024805. doi: 10.3389/fgene.2022.1024805 (PMC9638847; doi:10.3389/fgene.2022.1024805)
Supplement: Supplementary file 1 [file DataSheet1.docx]

**Supporting information**

**S1 Fig. Enriched GO terms of altered genes in MSUS Sertoli cells.** (a) Enriched GO pathways of significantly altered genes (p<0.05) in MSUS mouse Sertoli cells detected by RNA sequencing. Ratio of genes per pathway is given on the x-axis and log of p-value (log(p)) is indicated on a color scale. Bracket and arrow indicate mitochondrially related pathways. BP: biological process; CC: cellular component; MF: molecular function

**S2 Fig. High variability in mitochondrially encoded genes and mitochondrial CNV in batch 1 Sertoli cells, but high viability in all batch 1 samples.** (a) Heatmap of altered mitochondrially encoded genes of KEGG pathway “oxidative phosphorylation”. Fold change relative to controls is indicated in the color scale. (b) Fold changes in mitochondrial CNV of MSUS compared to control Sertoli cells of mouse batches 1 and 2. Error bars: mean ± SEM; ** = p<0.01, student’s t-test. (c) Heatmap of housekeeping genes (*Actb, Gapdh, Rplp0*) and cell death marker genes (*Casp9, Casp8, Casp7, Casp3*). Color scale indicates normalized log2 gene counts per million (CPM).

**S3 Fig. Characterization of lactate-to-pyruvate ratio and ROS production after MSUS serum exposure in primary Sertoli cells.** (a) Lactate and (b) pyruvate levels (in nmol/µl) in primary Sertoli cell medium after 24h of serum exposure. (c) Ratio of lactate to pyruvate and (d) ROS fluorescent signal in primary Sertoli cells after serum exposure. Controls, n=7; MSUS, n=9 for all graphs; error bars: mean ± SEM; ns=not significant, student’s t-test.

**S1 Table. Information on mice used for experiments.** (A) Mice used for RNA sequencing of Sertoli cells (Batch 1). (B) Mice used for Fluidigm RT-qPCR of Sertoli cells (Batch 2). (C) Mice used for serum collection for in vitro experiments. The tables contain information on the number (ID), group, cage, and litter cage.

**S2 Table. Counts of vimentin-positive cells in isolated FACS populations.** (A) Raw counts of each isolated fraction (before FACS, haploid, diploid, tetraploid, high FSC, low FSC, high APC and low APC) for 4 individual mice (4 replicates). Always at least 3 individual pictures per replicate were taken and counted. (B) Total number of cells counted for each replicate and each isolated fraction. (C) Percentages of vimentin positive cells out of all DAPI positive cells for each replicate and isolated fraction. Weighted average and weighted standard deviation (SD) were calculated from percentages.

**S3 Table. List of primer sequences.** The table includes information on the target genes, forward primer and reverse primer sequences.

**S4 Table. Summary of the over-representation analyses of significantly altered genes.** All significantly altered genes (p<0.05) in response to MSUS were used for over-representation analyses using Gprofiler. The table shows the source, term name, term ID and adjusted p-value for each significant pathway.
